# The prognostic significance of HDL-associated apolipoproteins in ascitic fluid from patients with cirrhosis and spontaneous bacterial peritonitis

**DOI:** 10.1038/s41598-025-08238-1

**Published:** 2025-07-16

**Authors:** Mohamad Murad, Philipp A. Reuken, Kristin Schubert, Johanna Reißing, Oluwatomi Ibidapo-Obe, Karsten Große, Mick Frissen, Frederic Haedge, Majda El-Hassani, Martin von Bergen, Tony Bruns

**Affiliations:** 1 Department of Internal Medicine III, University Hospital RWTH Aachen, Pauwelsstraße 30, 52074 Aachen, Germany; 2 Department of Internal Medicine IV, University Hospital Jena, Jena, Germany; 3 Department of Molecular Toxicology (MOLTOX), Helmholtz-Centre for Environmental Research (UFZ), Leipzig, Germany; 4 German Centre for Integrative Biodiversity Research (iDiv) Halle-Jena-Leipzig, Leipzig, Germany; 5 Institute of Biochemistry, Faculty of Life Sciences, University of Leipzig, Leipzig, Germany

**Keywords:** Cirrhosis, Ascites, HDL, Apolipoprotein A-II, Apolipoprotein A-I, Liver cirrhosis, Portal hypertension

## Abstract

Apolipoproteins (Apo) A-I and A-II are major components of high-density lipoproteins (HDL) with immunomodulatory functions. Low serum apoA-I levels indicate complications of cirrhosis. We hypothesized that HDL-associated apolipoproteins in ascitic fluid (AF) are differentially abundant during spontaneous bacterial peritonitis (SBP). Serum and non-infected AF samples from 18 patients with cirrhosis (cohort 1) were analyzed using LC–MS/MS to identify differentially regulated apolipoproteins. In cohort 2, samples from 59 patients with SBP and 59 without were analyzed for AF apoA-II and apoA-I concentrations using ELISA, and cumulative 90-day transplant-free survival was analyzed. Proteomic analysis indicated depletion of AF apoA-II in the absence of SBP. Lower AF apoA-II and apoA-I correlated with lower AF protein and albumin, higher serum bilirubin, and lower platelets. During SBP, AF apoA-II levels significantly increased, leading to higher apoA-II/apoA-I ratios. AF apoA-II, but not apoA-I, correlated with peritoneal IL-6 concentrations. AF apoA-II > 45 µg/ml during SBP indicated an increased risk of death or transplantation within 90 days and remained a predictor of transplant or death after adjustment for age and MELD (adjusted hazard ratio, 2.59; 95% CI, 1.08–5.91; P = 0.032). In summary, the composition of HDL-associated apolipoproteins in ascites is altered during SBP and correlates with disease course.

## Introduction

Patients with advanced cirrhosis are at an increased risk of bacterial infections, which significantly alter the natural course of the disease in both compensated and decompensated states^1–3^. Bacterial infections are increasingly recognized as precipitators of acute decompensation, often culminating in acute-on-chronic liver failure (ACLF), an inflammatory syndrome characterized by multiorgan failure and high short-term mortality^4,5^. Approximately one-third of all patients hospitalized for decompensated cirrhosis either have a bacterial infection upon admission or develop one during their stay^6,7^. The number of hospitalizations due to infections in cirrhosis increased by more than 50% from 2016 to 2018 in Germany, and infections were identified as an independent risk factor for in-hospital mortality with an odds ratio of 4.7^8^.

Spontaneous bacterial peritonitis (SBP) is a prototypical disease of the gut-liver axis^9,10^ accounting for approximately 25% to 30% of all bacterial infections in cirrhosis^6,7,11^ with an estimated lifetime risk of 28%^12^. The risk factors for SBP include advanced liver disease, dysbiosis, low ascitic fluid complement levels, intestinal bacterial overgrowth, proton pump inhibitor therapy, gastrointestinal bleeding, and genetic variants of innate immunity^13^. While annual admissions for SBP are on the rise, in-hospital mortality has peaked between 17 and 19% in the US^14^. In addition to the critical role of systemic dysfunctional immune responses in driving infection-mediated ACLF^15^, accumulating evidence suggests that impaired immune responses at the peritoneal level contribute to the susceptibility, course, and outcome of SBP in patients with cirrhosis^12,16–19^.

Recent studies have linked low high-density lipoprotein (HDL) and HDL-associated markers in plasma with complications of decompensated cirrhosis, including severe bacterial infections, organ failure, and mortality^20,21^. In addition to its major role in reverse cholesterol transport, HDL has crucial anti-inflammatory, antioxidant, and antimicrobial properties^22^. HDL is a heterogeneous population of lipoproteins defined by a density range of 1.063–1.21 g/ml^23,24^. Based on their density, two subclasses can be distinguished: HDL2 and HDL3^23^. HDL2 is the main type in systemic circulation. The smaller, denser subclass, HDL3, is primarily produced in the small intestine, is enriched in portal venous blood, and attenuates endotoxin-mediated immune responses in the liver^25^.

HDL-associated apolipoprotein A-I (apoA-I) is the main structural and functional protein, constituting approximately 70% of the total HDL protein, and lower plasma concentrations correlate with ACLF and mortality in decompensated cirrhosis^20^. Little is known about the relevance and prognostic significance of the second most abundant apolipoprotein in HDL, apolipoprotein A-II (ApoA-II), which accounts for approximately 20% of the total HDL protein^23,24^ and is enriched in anti-inflammatory human HDL3^26^.

We hypothesized that HDL-associated apolipoproteins in the ascitic fluid (AF) vary in abundance during SBP. Given that portal hypertensive ascites is lipoprotein-depleted and preferentially filters apolipoproteins by size influenced by endothelial permeability^27^, our study aimed to investigate concentrations of apoA-I and apoA-II in ascitic fluid, their changes during SBP, and evaluate their potential prognostic implications.

## Results

### Apo A-II is depleted from non-infected ascites

**Fig. 1:**
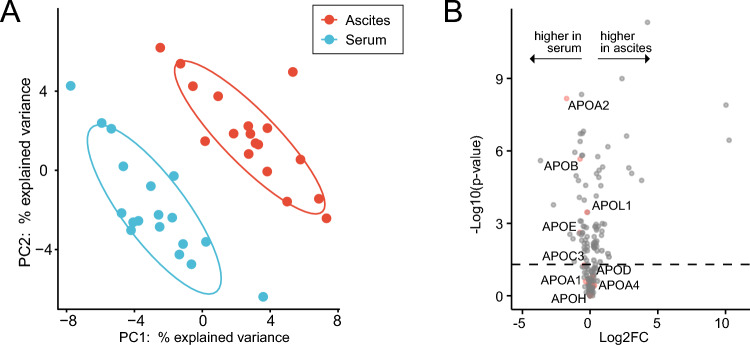
ApoA-II is depleted from uninfected ascites. (**A**) Principal component analysis (PCA) demonstrates the different protein profiles in serum (blue) and ascitic fluid (red) in patients with decompensated cirrhosis in the absence of spontaneous bacterial peritonitis (cohort 1, n = 18). (**B**) Volcano plot showing the regulation of apolipoproteins in serum and ascitic fluid showing the relationship between statistical significance and fold-change (FC) of protein expression in paired analysis. Points above the horizontal line indicates a statistical significance without correction for multiple testing (P < 0.05). Apolipoproteins are labelled and indicated in red.

To identify the apolipoprotein composition and its variation between serum and ascitic fluid in the absence of infection, we performed a global proteomic analysis of 18 patients with decompensated cirrhosis without SBP (cohort 1). Patients were predominantly male with alcohol-related cirrhosis and had advanced cirrhosis with a median Model for End-Stage Liver Disease (MELD) of 17 (Table 1). LC–MS/MS quantified 186 proteins. Principal component analysis (PCA) demonstrated distinct protein profiles between serum and AF (Fig. 1A). The following apolipoproteins were detected in both the serum and AF: apoA-I, apoA-II, apoA-IV, apoB, apoC-III, apoD, apoE, apoH, and apoL1. Among these, apoA-II was the apolipoprotein with the strongest relative depletion in AF compared to serum (Fig. 1B).

**Table 1:** Baseline characteristics (cohort 1, proteomics study).

	Total N = 18
Male sex	14 (78)
Age (years)	61 (57–66)
Alcohol-related liver disease	12 (67)
MELD score	17 (13–24)
Child pugh stage B/C	13 (72) / 5 (28)
Ascitic fluid characteristics	
AF leukocytes (/µl)	170 (130–370)
AF neutrophils (/µl)	30 (10–50)
AF protein (g/l)	15 (8–25)
AF albumin (g/l)	8 (4–15)
SAAG (g/l)	22 (19–25)
Serum characteristics and clinical chemistry	
Albumin (g/l)	30 (28–35)
Bilirubin (mg/dl)	1.5 (1.0–2.9)
International normalized ratio	1.5 (1.2–1.9)
Creatinine (mg/dl)	1.4 (0.8–2.3)
Sodium (mmol/l)	137 (133–139)
Platelets (/nl)	102 (82–168)
White blood cells (/nl)	5.2 (4.2–7.0)
C-reactive protein (mg/l)	18 (9–32)
ALT (U/l)	21 (18–38)
AST (U/l)	37 (31–64)

Data are expressed as medians with interquartile ranges (IQR) or as frequencies with percentages, as appropriate.

MELD, Model for End-Stage Liver Disease; AF, ascitic fluid; SAAG, serum ascites albumin gradient; ALT: Alanine transaminase; AST: Aspartate transaminase.

AF ApoA-II was significantly depleted from the ascitic fluid, resulting in approximately 30% of serum concentration (log_2_ fold change − 1.735; P < 0.001). In addition, apoE (log_2_ fold change − 0.748, P = 0.002) and apoL1 (log_2_ fold change − 0.235, P < 0.001) levels were lower in AF than in serum, albeit to a lesser extent. ApoA-I was not significantly regulated between the serum and AF (log_2_ fold change − 0.336; P = 0.25).

### Advanced liver disease is characterized by lower AF concentrations of apoA-II and apoA-I

Patients in cohort 2 were selected through a case–control design, comprising 59 individuals with SBP and 59 without SBP. Both cohorts were balanced for age, sex, and the serum ascites albumin gradient (Table 2). As expected, patients with SBP exhibited more advanced liver disease, a higher frequency of hepatic or extrahepatic organ failure, and a greater degree of systemic inflammation but without a significant difference in the degree of portal hypertension, as indicated by the platelet or serum ascitic fluid albumin gradient. Serum concentrations of total cholesterol, HDL cholesterol, and LDL cholesterol were lower in patients with SBP as compared to patients without while triglycerides were comparable. AF cholesterol levels remained unchanged during SBP. Despite the limitation that over 40% of concentrations were below the lower limit of detection of the automated assay used, we observed reduced AF LDL cholesterol concentrations in patients with SBP without changes in HDL cholesterol (Table 2).

**Table 2:** Baseline characteristics (cohort 2, prognostic study).

	Total N = 118	no SBP, N = 59	SBP, N = 59	P value
Male sex	91 (77)	46 (78)	45 (76)	1.00
Age (years)	60 (53–67)	62 (55–67)	58 (51–67)	0.20
Alcohol-related liver disease	83 (70)	47 (80)	36 (61)	0.043
Child–Pugh C	64 (54)	23 (39)	41 (70)	< 0.001
MELD score	18 (12–23)	16 (11–19)	21 (16–28)	< 0.001
ACLF (n (%))	34 (29)	9 (15)	25 (42)	0.002
ACLF Grade no/1/2/3	84 / 24 / 5 / 5	50 / 9 / 0 / 0	34 / 15 / 5 / 5	< 0.001
Ascitic fluid characteristics				
AF leukocytes (/µl)	470 (140–2010)	140 (80–230)	1990 (1110–4110)	< 0.001
AF neutrophils (/µl)	220 (20–1360)	20 (10–40)	1350 (400–2740)	< 0.001
AF protein (g/l)	13 (8–18)	13 (9–19)	13 (8–18)	0.41
AF albumin (g/l)	6 (5–10)	7 (5–11)	6 (5–10)	0.43
SAAG (g/l)	17 (13–22)	17 (14–22)	17 (11–22)	0.44
AF Total cholesterol (mg/dl)	18 (11–28)	19 (11–33)	16 (11–25)	0.37
AF Triglycerides (mg(dl)	32 (22–51)	41 (26–54)	26 (16–41)	< 0.001
AF HDL cholesterol (mg/dl)	3 (3–5)	3 (3–6)	3 (3–4)	0.14
AF LDL cholesterol (mg/dl)	5 (4–10)	6 (4–13)	4 (4–8)	0.024
Serum characteristics and clinical chemistry				
Albumin (g/l)	26 (21–31)	26 (21–32)	25 (19–30)	0.72
Bilirubin (mg/dl)	2.8 (1.3–5.2)	1.9 (1.1–4.4)	3.3 (1.8–9.4)	0.027
International Normalized Ratio	1.5 (1.2–1.7)	1.4 (1.2–1.6)	1.5 (1.3–2.0)	0.002
Creatinine (mg/dl)	1.2 (0.8–1.8)	0.9 (0.8–1.4)	1.2 (0.8–2.2)	0.021
Sodium (mmol/l)	135 (132–138)	136 (133–138)	135 (131–139)	0.49
Platelets (/nl)	122 (72–194)	138 (75–198)	108 (71–194)	0.64
White blood cells (/nl)	7.2 (4.9–11.2)	6.2 (4.2–8.9)	9.2 (6.2–15.1)	< 0.001
C-reactive protein (mg/l)	40 (22–82)	25 (14–44)	71 (39–123)	< 0.001
ALT (U/l)	30 (21–42)	29 (22–38)	30 (20–48)	0.76
AST (U/l)	55 (38–85)	48 (36–70)	57 (42–96)	0.12
Total cholesterol (mg/dl)	92 (53–130)	108 (84–154)	68 (39–92)	< 0.001
Triglycerides (mg(dl)	71 (55–99)	72 (57–102)	71 (53–94)	0.27
HDL cholesterol (mg/dl)	15 (8–24)	20 (14–27)	9 (5–14)	< 0.001
LDL cholesterol (mg/dl)	45 (19–72)	62 (44–96)	24 (8–45)	< 0.001
Cum. estimates of 90-days transplant-free survival (estimate, standard error)	53.8% (4.7%)	63.6% (6.4%)	43.7% (6.6%)	0.035*

Data are expressed as medians with interquartile ranges (IQR) or as frequencies with percentages, as appropriate. P-values from Fisher’s exact test for discrete data or Mann–Whitney U test for continuous data. Cumulative estimates of survival were obtained using Kaplan–Meier estimates.

MELD, Model for End-Stage Liver Disease; ACLF, acute-on-chronic liver failure; AF, ascitic fluid; SAAG, serum ascites albumin gradient; HDL high density lipoprotein; LDL low density lipoproteins; ALT, Alanine transaminase; AST, Aspartate transaminase. * log-rank test.

Cumulative estimates of 90-day transplant-free survival in patients without SBP were 63.6% (standard error 6.4%) compared to 43.7% (standard error 6.6%) in patients with SBP (P = 0.035 in log-rank test).

**Fig. 2:**
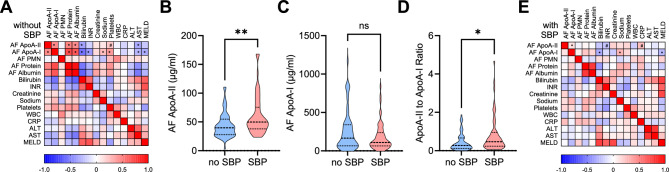
Ascitic fluid concentration of apoA-II in patients with decompensated cirrhosis with and without SBP. (**A**) Heatmap showing the nonparametric correlation (Spearman’s rho) of ascitic fluid (AF) concentrations of apoA-II and apoA-I in patients with cirrhosis without SBP (n = 59), with routine laboratory parameters and the MELD score. Significant correlations with AF apoA-II and AF apoA-I are indicated by asterisks. (B-D) Truncated violin plots of (**B**) AF apoA-II, (**C**) AF apoA-I, and (**D**) AF apoA-II-to-apoA-I ratio in patients without SBP (n = 59) and patients with SBP (n = 59). P value from Mann–Whitney U test. (**E**) Heatmap showing the nonparametric correlation (Spearman’s rho) of ascitic fluid (AF) concentrations of apoA-II and apoA-I in patients with cirrhosis with SBP (n = 59). *P < 0.05, #P < 0.10, **P < 0.01, ns: not significant. AF, ascitic fluid; apoA-II, apolipolipoprotein A-II; apoA-I, apolipoprotein A-I; PMN, polymorphonuclear neutrophils; INR, international normalized ratio; WBC, white blood cells; CRP, C-reactive protein; ALT, alanine transaminase; AST, aspartate transaminase; MELD, Model for End-Stage Liver Disease; SBP, spontaneous bacterial peritonitis.

In patients without SBP, the non-parametric correlation between apoA-II and apoA-I in AF was moderate (r_s_ = 0.460, P < 0.001), and AF apoA-II and apo apoA-I did not significantly correlate with serum triglycerides, total serum cholesterol, or serum LDL cholesterol. While AF apoA-II did not correlate significantly with serum HDL cholesterol, it showed a moderate positive correlation with AF total cholesterol (r_s_ = 0.452, P < 0.001), and weak positive correlations with AF HDL cholesterol (r_s_ = 0.278, P = 0.033) and AF LDL cholesterol (r_s_ = 0.332, P = 0.010). AF apoA-I correlated positively with serum HDL cholesterol (r_s_ = 0.384, P = 0.004), showed a strong positive correlation with AF total cholesterol (r_s_ = 0.736, P < 0.001) and AF LDL cholesterol (r_s_ = 0.642, P < 0.001), and a moderate positive correlation with AF HDL cholesterol (r_s_ = 0.582, P < 0.001). Lower AF apoA-II levels correlated with lower AF protein (r_s_ = 0.483, P < 0.001), reduced AF albumin (r_s_ = 0.467, P < 0.001), and higher serum bilirubin (r_s_ = − 0.364, P = 0.005), with a non-significant trend towards correlation with platelets (r_s_ = 0.219, P = 0.096). AF apoA-I correlated positively with AF protein (r_s_ = 0.721, P < 0.001), AF albumin (r_s_ = 0.655, P < 0.001), platelets (r_s_ = 0.403, P = 0.002), and sodium (r_s_ = 0.261, P = 0.046), but negatively correlated with serum bilirubin (r_s_ = − 0.554, P < 0.001), INR (International Normalized Ratio) (r_s_ = − 0.399, P = 0.002), and the MELD score (r_s_ = − 0.332, P = 0.010). No significant correlations were found between renal function or systemic inflammation markers (white blood cells [WBCs] and C-reactive protein [CRP]) and AF apolipoproteins in patients without SBP (Fig. 2A). In addition, AF apoA-II concentrations did not differ significantly between males and females.

### SBP is characterized by an enrichment of apoA-II in ascitic fluid, resulting in an elevated apoA-II/A-I ratio

AF concentrations of apoA-II were higher in patients with SBP than in patients without (median concentration 49.7 vs 39.8 µg/ml, P = 0.001, Fig. 2B). In contrast to ApoA-II, apoA-I was not increased in patients with SBP (median 108.8 vs. 165.7 µg/ml, P = 0.23) resulting in a higher AF apoA-II/apoA-I ratio in patients with SBP as compared to patients without (Fig. 2C,D). In SBP, the previously observed correlations of AF apoA-II with ascitic protein, albumin, serum bilirubin, and platelets were diminished (Fig. 2E). AF apoA-II exhibited only a weak and non-significant association with CRP (rs = 0.247, P = 0.059) and INR (rs = − 0.231, P = 0.079), while AF apoA-I retained its correlation with serum bilirubin (rs = − 0.322, P = 0.013), MELD score (rs = − 0.273, P = 0.036), and serum sodium (rs = 0.263, P = 0.004) (Fig. 2E).

Similar to patients without SBP, AF apoA-II did not correlate with serum cholesterol or triglyceride levels but with AF total cholesterol (r_s_ = 0.465, P < 0.001), AF HDL cholesterol (r_s_ = 0.331, P = 0.015), and AF LDL cholesterol (r_s_ = 0.444, P < 0.001). AF ApoA-I also correlated with AF total cholesterol (r_s_ = 0.482, P < 0.001), AF HDL cholesterol (r_s_ = 0.468, P < 0.001), AF LDL cholesterol (r_s_ = 0.528, P =  < 0.001).

### Enrichment of apoA-II in AF indicates higher peritoneal IL-6

**Fig. 3:**
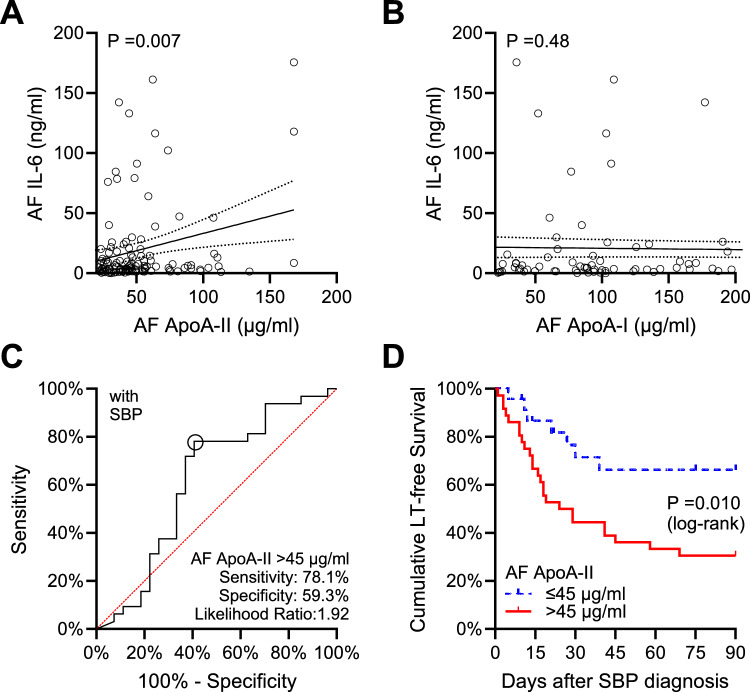
AF apoA-II correlates with AF IL-6 and predicts transplant-free survival in patients with SBP. (A-B) Scatter plot with linear regression of (**A**) AF apoA-II and (**B**) AF apoA-I with AF IL-6 concentrations. P value from Pearson correlation is indicated. (**C**) Receiver operating characteristics (ROC) for AF apoA-II to predicted death or the necessity for liver transplantation within 90 days in patients with SBP (n = 59). Optimum cut-off according to the Youden index is indicated (circle) and corresponding sensitivity, specificity and likelihood ratio are shown. (**D**) Kaplan–Meier analysis of cumulative 90-day transplant-free survival in patients with SBP stratified into two groups based on the AF apoA-II cutoff. P value from Log-rank test is shown. AF, ascitic fluid; apoA-II, apolipolipoprotein A-II; IL-6, interleukin-6; apoA-I, apolipolipoprotein.

To assess the correlation between HDL-associated apolipoproteins and peritoneal inflammation, we correlated AF apolipoproteins with IL-6 in ascites as a marker of peritoneal macrophage activation^12,28^. Although there was large inter-individual variability in AF IL-6 concentrations, higher AF apoA-II concentrations correlated with higher AF IL-6 levels (Pearson’s r = 0.25, P = 0.007), while there was no correlation between AF apoA-I and AF IL-6 (Pearson’s r = 0.07, P = 0.48) (Fig. 3A,B).

### Higher AF apoA-II concentrations indicate patients with poor outcomes of SBP

To assess whether higher AF apoA-II concentrations during SBP indicated a poor outcome, we assessed its association with short-term transplant-free survival. In the cohort of patients with SBP, 28 (47%) died, and 4 (7%) underwent liver transplantation within 90 days. Four patients (7%) were lost to follow-up after a median follow-up of 41 days.

Although the overall predictive ability of AF apoA-II for poor outcomes was only moderate, AF concentrations exceeding 45 μg/ml predicted death or the necessity for liver transplantation within 90 days, with a sensitivity of 78% and a specificity of 59% (Fig. 3C). Time-to-event analysis comparing Kaplan–Meier estimates of 90-days transplant-free survival confirmed lower transplant-free 90-days survival (estimate: 33%, standard error: 8%) in patients with AF apoA-II > 45 μg/ml than in patients with AF apoA-II of 45 μg/ml or below (estimate: 66%, standard error: 10%, P = 0.010) (Fig. 3D).

Univariate Cox regression analysis of transplant-free survival was performed identifying parameters affecting prognosis in patients with SBP, including age, the MELD score, Child–Pugh stage, the presence of ACLF, and inflammatory biomarkers. Parameters which were significant in univariate analysis (higher apoA-II, presence of ACLF, older age) and are well known to affect prognosis of patients with SBP (MELD) were assessed in three alternative multivariable Cox regression models (Table 3). In all models AF apoA-II > 45 μg/ml during SBP remained a predictor of transplant or death after adjustment for the presence of acute-on-chronic liver failure (ACLF) grade 2/3 alone (model 1, adjusted hazard ratio [HR] 2.89; 95% confidence interval [CI]: 1.25–6.71; P = 0.01), age and ACLF grade 2/3 (model 2, adjusted HR 2.59; 95% CI: 1.11–6.04; P = 0.03), or for age and MELD score (model 3, adjusted HR 2.53; 95% CI: 1.08–5.91; P = 0.03) (Table 3).

**Table 3:** Multivariable Cox regression analysis for transplant or death within 90 days after SBP.

	Univariate analysis			Multivariable model 1			Multivariable model 2			Multivariable model 3		
	HR	CI	P value	HR	CI	P value	HR	CI	P value	HR	CI	P value
AF apoA-II > 45 µg/ml	2.840	1.227–6.573	0.015	2.891	1.247–6.706	0.013	2.587	1.108 -6.041	0.028	2.529	1.083–5.908	0.032
MELD > 22	1.588	0.788–3.201	0.196	Not included			Not included			Not included		
MELD (per point increase)	1.031	0.990–1.074	0.144	Not included			Not included			1.047	0.997–1.099	0.064
Child–Pugh C (vs. B)	2.630	0.360–19.236	0.341	Not included			Not included			Not included		
Age (per 1-year increase)	1.049	1.020–1.080	0.001	Not included			1.046	1.015–1.077	0.003	1.053	1.020–1.088	0.002
Male sex	0.851	0.455–1.591	0.613	Not included			Not included			Not included		
White blood cells (per 1/nl increase)	1.003	0.971–1.035	0.877	Not included			Not included			Not included		
ACLF yes/no	1.736	0.864–3.489	0.122	Not included			Not included			Not included		
ACLF grade 2/3	2.447	1.080–5.543	0.032	2.533	1.107–5.795	0.028	2.310	0.997–5.353	0.051	Not included		
C-reactive protein (per 1-mg/l increase)	1.005	0.999–1.010	0.124	Not included			Not included			Not included		
AF IL-6 (per 1-ng/ml increase)	1.000	0.993–1.008	0.918	Not included			Not included			Not included		

AF, ascitic fluid; apoA-II, apolipoprotein A-II; MELD, Model for End-Stage Liver Disease; ACLF, acute-on-chronic liver failure; IL-6, interleukin 6; HR, hazard ratio; CI, confidence interval.

## Discussion

Reduced levels of circulating HDL-linked biomarkers, specifically apoA-I and HDL cholesterol, are linked to more advanced liver disease, systemic inflammation, infectious complications, and organ failure in cirrhosis with an increased risk of mortality^20^. Here, we demonstrate that in patients with decompensated cirrhosis, the HDL-related apolipoprotein apoA-II was depleted from non-infected ascites and exhibited marked changes during SBP, which was associated with poor outcomes in this cohort study.

In cirrhotic ascites, molecular size and portal pressure determine lower HDL and thus apolipoprotein concentrations. However, conditions that cause endothelial leakage, such as peritoneal carcinomatosis, can significantly affect peritoneal HDL concentration^27^. Proteomic analysis of paired serum and ascites samples revealed a significant depletion of apolipoproteins in ascites, with apoA-II showing the strongest reduction. This suggests a predominance of apoA-I-rich and apoA-II-poor HDL in uninfected ascites, distinct from that found in blood^29^. We observed a correlation between declining levels of both apoA-I and apoA-II in non-infected ascites and advanced liver disease. This correlation was evident through associations with AF protein, albumin, serum bilirubin, and platelets, suggesting that the concentrations of AF apoA-I and apoA-II may indicate deteriorating liver function.

ApoA-I and apoA-II are primarily synthesised in the liver and intestines and act as negative acute-phase proteins^30,31^. Consequently, patients with bacterial infections experience decreased levels of HDL-associated apolipoprotein A-I and a significant reduction in HDL-associated cholesterol in the serum^32^. During SBP, an increase in the AF apoA-II to apoA-I ratio was noted in our study, which could be indicative of a relevant change in HDL diameter and composition in ascites or the presence of lipid-poor or lipid-free forms of apo-A-II. While serum concentrations of total and HDL-cholesterol were decreased during an episode of SBP, we did not observe such a decrease in ascitic fluid, albeit limited by very low peritoneal AF cholesterol levels. Unexpectedly, elevated AF apo-AII concentrations during SBP were associated with an increased risk for adverse outcomes. We hypothesized that this peritoneal increase is due to an augmented bloodstream influx driven by enhanced endothelial permeability, which significantly alters the peritoneal lipoprotein landscape^33^. Although significant correlations were not found with systemic (WBC, CRP) or peritoneal inflammation markers (AF leukocytes, AF polymorphonuclear neutrophils), a moderate correlation between AF apoA-II concentrations and IL-6 was observed. This suggests increased activation of peritoneal macrophages in the presence of higher levels of AF apoA-II, with potential prognostic relevance^16,18^.

Beyond its role as a surrogate for increased endothelial permeability during SBP, an increased apoA-II to apoA-I ratio may also directly impact the metabolic and immunomodulatory functions of HDL and its subclasses in the peritoneal cavity. Melchior et al. proposed an elegant model suggesting that apoA-II content predominantly determines the size and metabolic activity of apoA-I-containing HDL^34^. ApoA-II causes structural changes, enhances cholesterol efflux^35^, and affects the release of fatty acids from HDL2 and HDL3^36^. Peritoneal changes in HDL concentration and composition have immunomodulatory potential^22^. Reconstituted HDL ameliorates excessive immune responses of monocytes from patients with cirrhosis^37^ and reduces hepatic inflammation and portal pressure in cirrhotic rats^38^. HDL3 protects the liver from injury to enterically derived lipopolysaccharides (LPS) in portal circulation when complexed with LPS-binding protein (LBP)^25^.

The immunomodulatory role of apoA-II remains controversial. In contrast to apoA-I and other apolipoproteins, purified apoA-II does not directly inhibit LPS bioactivity as it suppresses LBP function^39^. In contrast, apoA-II has been reported to inhibit the phosphorylation of ERK1/2 (extracellular signal-regulated kinase 1/2) and transcription factor Jun (c-Jun)^40^, which is a hallmark of activated peritoneal macrophages in patients with cirrhosis and correlates with augmented IL-6 secretion^41^. As a result, the influx of apoA-II- and apoA-II-rich HDL may exert pro-inflammatory functions that are dependent on the local availability of LBP and competing apolipoproteins during SBP.

Our study has limitations. Serum concentrations of apoA-I and apoA-II were not available in cohort 2. We can speculate about the variation of protein content within HDL being modulated by SBP as we did not quantify HDL particles within ascitic fluid or purified HDL from ascitic fluid to assess the protein composition. In addition, whether lipid-poor or lipid-free apolipoproteins have contributed to the observed changes in the total apoA-I/apoA-II ratio remains to be assessed by future studies.

In conclusion, we demonstrated compartmental differences in the abundance of essential HDL-associated apolipoproteins in the ascitic fluid of patients with decompensated cirrhosis. To our knowledge, this is the first study to demonstrate the distribution of the main HDL-associated apolipoproteins in cirrhotic inflamed ascites from SBP patients. In addition to being a surrogate biomarker for more severe courses of peritonitis, elevations in AF apoA-II concentrations during peritoneal inflammation may significantly alter HDL heterogeneity and composition and/or the availability of lipid-poor or lipid-free forms of apoA-II, potentially influencing immune surveillance and inflammation. Future studies should perform serial measurements of HDL size heterogeneity and protein content to confirm the composition alterations of HDL and investigate the immunomodulatory effects of isolated peritoneal lipoproteins and their potential as therapeutic targets.

## Patients and methods

### Patient cohorts

This was a retrospective analysis of paired serum and AF samples from two cohorts comprising an exploratory cohort study of 18 patients with decompensated cirrhosis and uninfected ascites (cohort 1) and a prognostic case–control study of 118 patients with decompensated cirrhosis and ascites, of which 59 had SBP and 59 did not have SBP (cohort 2). Patients were recruited from two German tertiary centers: Jena University Hospital and University Hospital RWTH Aachen. The included patients were hospitalized for complications of cirrhosis and underwent diagnostic tap or therapeutic paracentesis. Patients with peritoneal carcinomatosis or secondary peritonitis were excluded from this study. Serum and AF samples were processed as described previously^16,42^ and frozen at − 80 °C until analysis.

Demographic and clinical data, including the etiology and history of cirrhosis, were assessed at the beginning of the study. Clinical chemistry parameters were determined using routine laboratory tests. Triglycerides, total cholesterol, HDL cholesterol, and LDL cholesterol were directly measured in serum and ascitic fluid using enzymatic colorimetric tests on a Roche Cobas C701 Chemistry Analyzer designed for (Grenzach-Wyhlen, Germany).

The study was conducted according to local regulations and guidelines and was approved by the Internal Review Board of the Jena University Hospital (Ethics committee of the Jena University Hospital, no. 3683-02/3, 2019-1510-BO) and the Internal Review Board of the Medical Faculty of the RWTH Aachen (Ethik-Kommission der Medizinischen Fakultät RWTH Aachen, no. EK 327/19). Written informed consent was obtained from patients or their legal representatives. Patients were followed up for 90 days, with the primary outcomes being death, liver transplantation, or survival within 90 days.

### Proteomics

Serum and ascitic fluid samples from cohort 1 were diluted 1:10 in lysis buffer (8 M urea and 10 mM Dithiothreitol, both Sigma-Aldrich, Taufkirchen, Germany) and enzymatically cleaved with trypsin (Promega, Walldorf, Germany) at a 1:20 (trypsin:protein) ratio using paramagnetic beads. Peptides were eluted in 2% dimethyl sulfoxide (Sigma-Aldrich, Taufkirchen, Germany), resulting in one fraction that was analyzed by Liquid Chromatography Tandem Mass Spectrometry (LC–MS/MS) using an Ultimate 3000 nano ultra-performance liquid chromatography system (UPLC, Dionex, USA) coupled to a Q Exactive HF (Thermo Fisher Scientific, Waltham, USA). First, peptides were separated with a trapping column (flow rate 5 ml/min, Acclaim PepMap 100 C18, 3 µM, nanoViper, 75 µm × 5 cm, Thermo Scientific, Germany) and an analytical column (Acclaim PepMap 100 C18, 3 µm, nanoViper, 75 µm × 25 cm, Thermo Scientific, Germany) using a 80-min non-linear gradient of hydrophilic solution A (0.1% formic acid (v/v) in ddH2O) and hydrophobic solution B (80% acetonitrile (ACN; Merck, Darmstadt, Germany) and 0.1% ammonium formate (FA; Sigma Aldrich, Taufkirchen, Germany) in ddH2O, v/v). The raw data were processed against the UniProtKB reference proteome of Homo sapiens (6 December 2021), using MaxQuant 1.6.2.10 default settings with label-free quantification and match between runs allowed. All reverse and “only identified only by site” entries and potential contaminants were removed. The proteomic data were log2-transformed, median-normalized, and filtered for proteins found in at least nine replicates for each ascites and serum using the Proteomicsr package^43^. Log2 fold-changes were calculated, and significantly altered proteins were determined using a paired t-test without correction for multiple testing.

### Enzyme-linked immunoassay (ELISA)

AF apoA-II was measured using the APOA2 Human ELISA Kit (Abnova; KA0461) at a dilution of 1/5, following the manufacturer’s instructions. AF apoA-I was measured using the human apolipoprotein A-I Quantikine ELISA Kit (R&D Systems; DAPA10) at a dilution of 1/4000. AF IL-6 (interleukin 6) was measured in ascites using the IL-6 Human Matched Antibody Pair (Invitrogen; CHC1263) with a dilution of 1/50 in the absence of SBP and 1/80 in the presence of SBP. All ELISAs were evaluated using a Cytation3 multi-mode microplate reader (BioTek, Winooski, VT, USA).

### Statistical analysis

Statistical analyses were performed using IBM SPSS Version 29.0.0, and figures were generated using Prism 10.01 (GraphPad, San Diego, CA, USA). Fisher’s exact test and Mann–Whitney U test were used for between-group comparisons for categorical and continuous variables, respectively. For correlation analysis, nonparametric correlation was determined using Spearman’s correlation, while parametric correlation was conducted using Pearson’s correlation. Receiver operating characteristic (ROC) curve analysis and the Youden index were used to establish the optimal cutoff value for apoA-II concentration in AF. Kaplan–Meier analysis and multivariable Cox regression models assessed cumulative transplant-free survival within 90 days of SBP. Liver transplantation or death was counted as an event, whichever occurred first. The patients were right-censored at the time of loss to follow-up or at 90 days. All statistical tests were two-sided at a significance level of < 0.05, without adjustment for multiple testing.

## Data Availability

The datasets generated and analysed during the current study available from the corresponding author on reasonable request.

## Abbreviations

ACLF: Acute-on-chronic liver failure
apo: Apolipoprotein
AF: Ascitic fluid
CI: Confidence interval
c-Jun: Transcription factor Jun
CRP: C-reactive protein
ELISA: Enzyme-linked immunosorbent assay
ERK1/2: Extracellular signal-regulated kinase 1/2
HDL: High-density lipoprotein
IL-6: Interleukin 6
INR: International Normalized Ratio
LBP: Lipopolysaccharide binding protein
LDL: Low-density lipoprotein
LPS: Lipopolysaccharides
MELD: Model for End-Stage Liver Disease
PCA: Principal component analysis
ROC: Receiver operating characteristic
RWTH: Rheinisch-Westfälische Technische Hochschule
SBP: Spontaneous bacterial peritonitis
WBC: White blood cells
